# Differential Circulating MicroRNA Expression in Age-Related Macular Degeneration

**DOI:** 10.3390/ijms222212321

**Published:** 2021-11-15

**Authors:** Hanan ElShelmani, Ian Brennan, David J. Kelly, David Keegan

**Affiliations:** 1 Mater Misericordiae University Hospital, Eccles St., Dublin 7, Ireland; elshelh@tcd.ie (H.E.); ibrennan@rcsi.ie (I.B.); 2 University College Cork, College Road, Cork, Ireland; 3 Zoology Department, School of Natural Sciences, Trinity College Dublin, University of Dublin, Dublin 2, Ireland; djkelly@tcd.ie

**Keywords:** microRNA, age-related macular degeneration, biomarkers, serum, dry AMD, wet AMD

## Abstract

This study explored the expression of several miRNAs reported to be deregulated in age-related macular degeneration (AMD). Total RNA was isolated from sera from patients with dry AMD (*n* = 12), wet AMD (*n* = 14), and controls (*n* = 10). Forty-two previously investigated miRNAs were selected based on published data and their role in AMD pathogenesis, such as angiogenic and inflammatory effects, and were co-analysed using a miRCURY LNA miRNA SYBR^®^ Green PCR kit via quantitative real-time polymerase chain reaction (qRT-PCR) to validate their presence. Unsupervised hierarchical clustering indicated that AMD serum specimens have a different miRNA profile to healthy controls. We successfully validated the differentially regulated miRNAs in serum from AMD patients versus controls. Eight miRNAs (hsa-let-7a-5p, hsa-let-7d-5p, hsa-miR-23a-3p, hsa-miR-301a-3p, hsa-miR-361-5p, hsa-miR-27b-3p, hsa-miR-874-3p, hsa-miR-19b-1-5p) showed higher expression in the serum of dry AMD patients than wet AMD patients and compared with healthy controls. Increased quantities of certain miRNAs in the serum of AMD patients indicate that these miRNAs could potentially serve as diagnostic AMD biomarkers and might be used as future AMD treatment targets. The discovery of significant serum miRNA biomarkers in AMD patients would provide an easy screening tool for at-risk populations.

## 1. Introduction

Age-related macular degeneration (AMD) is the most common cause of blindness in people over 60 years of age [1,2,3]. AMD is a progressive retinal disease that can broadly be categorised into either “dry” atrophic AMD or “wet” neovascular AMD. Atrophic AMD is characterised in its early stages by dysfunction of the retinal pigment epithelium (RPE) along with the formation of drusen in Bruch’s membrane [4,5]. These changes result in atrophy and damage to the photoreceptor cells and RPE which in turn results in a slow, progressive, and irreversible loss of vision [4,5]. Geographic atrophy of the retina with corresponding significant visual deficit is seen in late-stage atrophic AMD [2,6]. Neovascular AMD is another form of late-stage disease, where loss of vision is attributable to the formation of new vessels within and below the retina via choroidal neovascularisation [2,6,7]. These new vessels are disorganised, friable and prone to haemorrhage, aberrant fibrovascular scarring and detachment, and RPE [1,2,5,7]. As a result, the progression of visual loss is markedly more rapid in neovascular AMD compared to atrophic AMD [2,6,8].

AMD has an insidious clinical onset, and as yet there are no effective means of screening for the disease [9,10]. Moreover, fundoscopy, imaging and self-monitoring for disease progression fall short of identifying patients who will go on to develop neovascular disease [9]. Therefore, there is a distinct need for the identification of useful AMD biomarkers that could be used in the diagnosis and recognition of disease progression.

MicroRNAs (miRNAs) are small noncoding RNA molecules involved in post-translational regulation of gene expression through the targeting and silencing of complementary messenger RNA (mRNA) [1,10,11]. Gene silencing by miRNA is thought to play a role in controlling a variety of both physiological and pathological processes [12]. It has also been shown that miRNA expression changes with ageing, and miRNAs that are normally downregulated by ageing remain unusually normal or become elevated in patients with AMD [10,13].

Indeed, miRNAs have been shown to have a governing function in processes underpinning AMD, such as inflammation, angiogenesis, and oxidative stress responses [12,14,15]. Additionally, AMD is a neurodegenerative disease [16] and there is interest in the commonality of some miRNAs expressed in AMD and in other neurodegenerative diseases, such as Alzheimer’s disease [4,15,17]. Their distinctive expression in these disease-states and their relative stability in serum samples make miRNAs very promising diagnostic biomarkers, and potential therapeutic targets [4,9,18,19,20].

Numerous clinical studies have investigated the differential expression of miRNAs in the serum of patients with AMD (Table 1). This study aims to validate a number of promising serum miRNA biomarkers identified in AMD, and to characterise their expression in the context of Irish patients with AMD.

Mature miRNAs are derived from the 3′ and 5′ ends of the same precursor. The pre-miRNA hairpin is cleaved by the RNase III enzyme Dicer to create mature miRNA from the two arms of the duplex. These mature miRNAs have two nucleotides overhanging on their 3′/5′ ends (miRNA-3p and miRNA-5p, respectively). It is known that, in some cases, both miRNA-5p and miRNA-3p are functional and target different RNA populations [36,37,38], though the selection of 3p/5p arm preference remains unclear [38]. This may account for some of the dual effects noticed in a number of studies under different biological conditions [14,24]. For example, miR-410 was discovered by ElShelmani et al. and Ertekin et al. to be dysregulated in AMD patients. It has two mature forms: miR-410-3p (sequence: AAUAUAACACAGAUGGCCUGU) and miR-410-5p (sequence: AGGUUGUCUGUGAUGAGUUCG) [10,21,39,40].

The objective of this study was to explore the expression of several miRNAs reported to be deregulated in AMD. Such differentially expressed serum miRNA could be used as AMD biomarkers. Furthermore, our study focused on finding the exact mature form of miRNAs that are overexpressed in the serum of AMD patients. Having selected relevant miRNAs from the literature, we were able to utilise miRbase (http://www.mirbase.org/, accessed on 1 February 2021) to find the mature form of each miRNA selected in this project [39,40].

## 2. Results

### 2.1. AMD miRNA Biomarkers, Data Quality Control, and Normalisation

**Figure 1:**
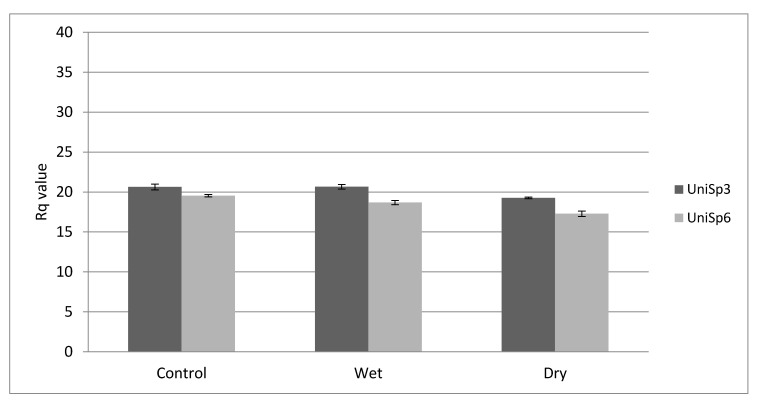
Raw Rq value (mean ± SD) obtained for the RNA spike-in assay. The steady level of the assays indicates that both RT and qRT-PCR were successful (*n* = 36). Dry = atrophic AMD, Wet = neovascular AMD.

The steady levels of the assay shown in Figure 1 indicate that both reverse transcription and qRT-PCR were successful. RNA spike-in control UniSp6 expression level indicates that the reverse transcription was also successful. UniSp3 indicates good technical performance of the qPCR (Figure 1).

miR-451a and miR-23a-3p are relatively stable in serum and plasma and are not affected by haemolysis. ΔRq (miR-23a, miR-451a) lower than 5 in human serum or plasma represents non-haemolyzed samples. If the ∆Rq is close to or higher than 7, there is an increased risk of haemolysis. In this study, any samples that showed ∆Rq higher than 7 were excluded (Figure S1). The ∆Rq for UniSp2, UniSp4, UniSp5 RNA Spike-in were 2–3 Rq difference within a dataset. Any sample showed higher differences of ∆Rq were excluded from the study (Figure S1).

**Figure 2:**
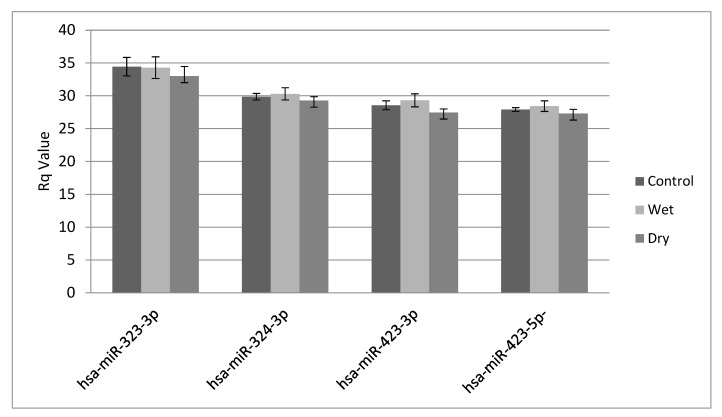
Raw Rq obtained for the four control miRNA assays used for normalisation (ANOVA: *p* = 0.132). Dry = atrophic AMD, Wet = neovascular AMD. miR-324-3p (*p* = 0.172), miR-423-3p (*p* = 0.201), and miR423-5p (*p* = 0.168) were used for normalization in this study.

Normalisation was performed based on the average of three miRNAs detected in all specimens (miR-324-3p, miR-423-3p and miR-423-5p) (Figure 2). miR-323-3p was excluded from the normalization as it showed higher values than the three other endogenous controls (Figure 2).

### 2.2. Differentially Expressed miRNAs in AMD

**Figure 3:**
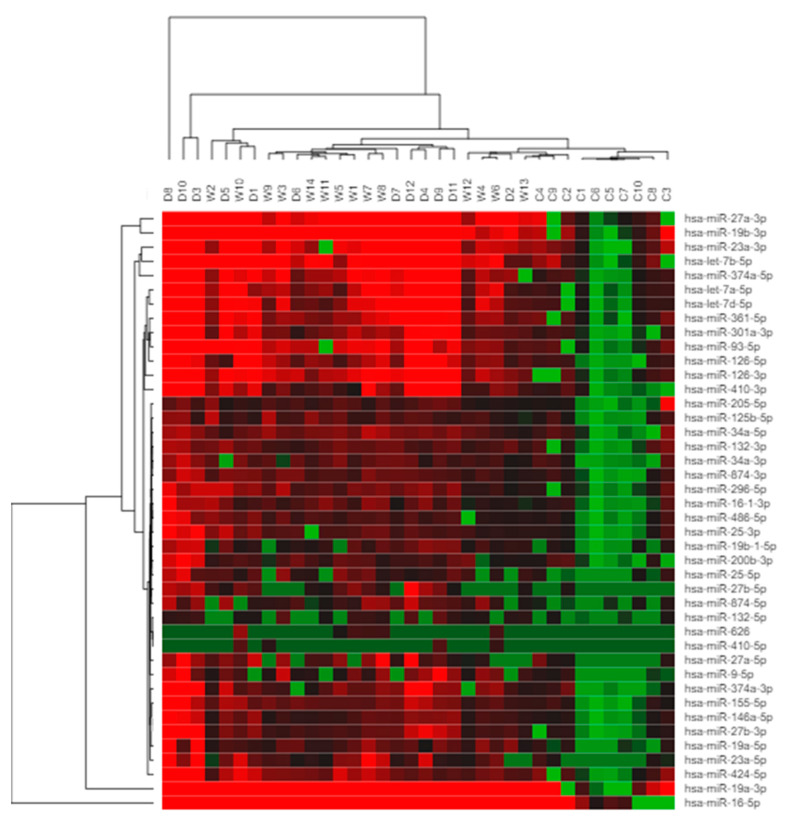
Hierarchical clustering of differentially regulated miRNAs. The clustering map represents miRNA differential expression in AMD patients and controls. Each row represents one miRNA, and each column represents one sample (dry, wet and control). Red = expression level above the mean, green = expression lower than the mean, (D1, D2, ..., D12 = dry AMD specimens; W1, W2, ..., W14 = wet AMD specimens; C1, C2, ..., C10 = control).

Hierarchical clustering analysis (Figure 3) performed for the study, using Morpheus software (https://clue.io/morpheus, accessed on 1 April 2021), showed the differential expressions of the miRNA candidates in wet and dry AMD patients groups compared with controls. Red and green colours indicate high- and low-expression intensities. Table 2 shows the individual results for these miRNAs.

### 2.3. MicroRNAs as Candidate Biomarkers for AMD

Thirty-nine up-regulated miRNAs (Table 2, Figure 3) were confirmed to be overexpressed in the serum of AMD patients. Post-hoc pairwise comparisons were made of the dry vs. control samples and wet vs. control samples. Eight miRNAs (hsa-let-7a-5p, hsa-let-7d-5p, hsa-miR-23a-3p, hsa-miR-301a-3p, hsa-miR-361-5p, hsa-miR-27b-3p, hsa-miR-874-3p, and hsa-miR-19b-1-5p) showed higher expression in the serum of dry AMD patients compared with healthy controls than in wet AMD patients compared with healthy controls.

## 3. Discussion

**Figure 4:**
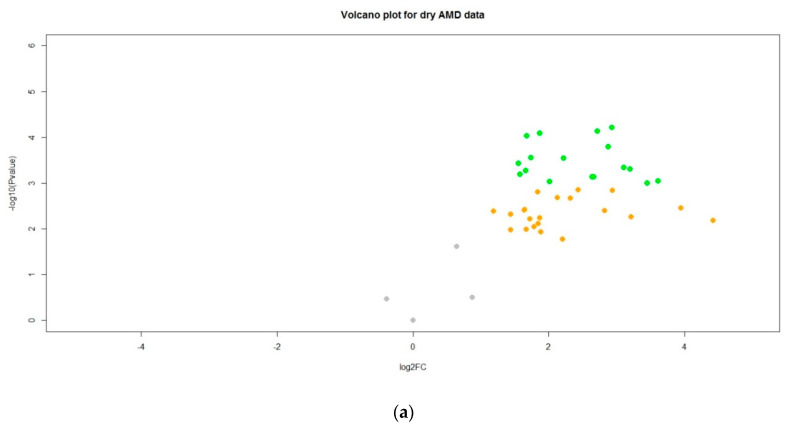

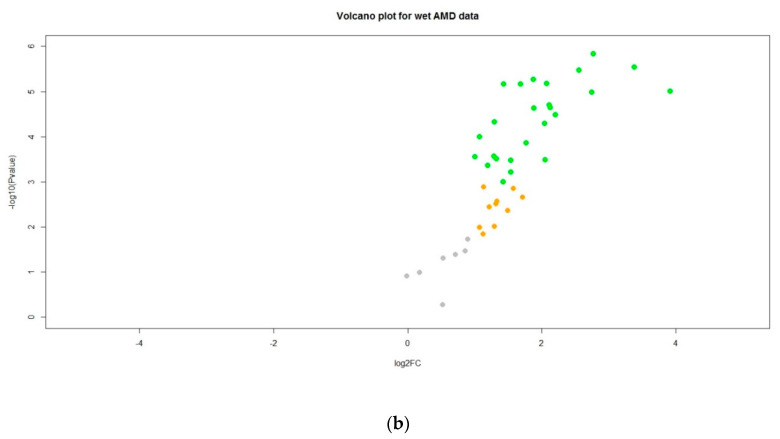
Volcano plots to show miRNA changes in (**a**) patients with ‘dry’ AMD and (**b**) patients with ‘wet’ AMD. Points are grey if there is neither a noticeable fold change in gene production nor a difference in gene expression between the control group and the AMD groups, after adjusting for multiple tests. Points are orange if there is a noticeable fold change in gene production, but no difference in gene expression between the control group and the AMD groups, after adjusting for multiple tests. Points are green if there is a noticeable fold change in gene production and a difference in gene expression between the control group and the AMD groups, after adjusting for multiple tests.

**Figure 5:**
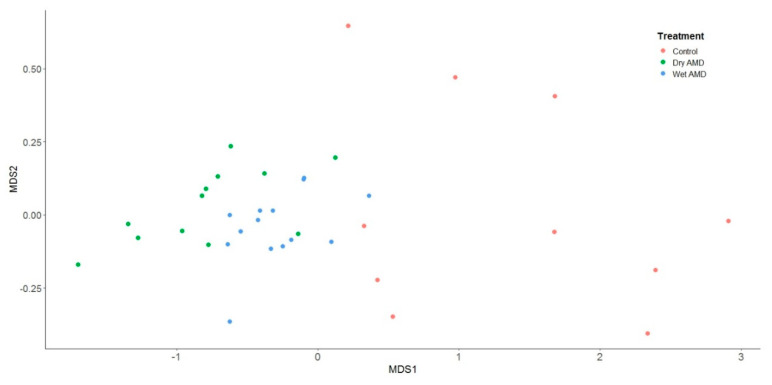
A multidimensional scaling plot of RQ values for each of the miRNAs under investigation from control patients (orange circles), patients with ‘dry’ AMD (green circles), and patients with ‘wet’ AMD (blue circles). Patients with both ‘dry’ and ‘wet’ AMD provided consistently lower values on the MDS1 axis (equivalent to upregulation of miRNAs). An analysis of variance for the distance matrix (performed by the *adonis* function within the vegan package in R) demonstrated a significant difference between patient groups (F(2,33) = 12.462, *p* < 0.01).

Our study successfully profiled the differential quantities of miRNAs in serum from AMD patients compared with healthy controls in an Irish context. Markedly different miRNA expression profiles were identified between the two groups. Of the candidate miRNAs selected for examination, 39 were significantly up-regulated in the serum of AMD patients compared with controls, validating their usefulness as potential biomarkers for AMD in Irish patients (Table 2, Figure 4). On MDS analysis of RQ values for each of miRNA, patients with both ‘dry’ and ‘wet’ AMD exhibited consistently raised expression levels of candidate miRNAs (Figure 5). Additionally, patients with AMD appear to cluster together, underlining the relative closeness in fold change of miRNA expression in these patients. Controls on the other hand, can be seen to be dispersed. Moreover, eight miRNAs identified showed higher expression in the serum of dry AMD patients than in that of wet AMD patients compared with healthy controls: hsa-let-7a-5p, hsa-let-7d-5p, hsa-miR-19b-1-5p, hsa-miR-23a-3p, hsa-miR-27b-3p, hsa-miR-301a-3p, hsa-miR-361-5p, and hsa-miR-874-3p. The differences between patients with dry AMD and wet AMD lends promise to the potential prognostic value of miRNA biomarkers.

The miRNAs identified in this study have been previously shown to play important roles in the regulation of AMD pathogenesis. Let-7 family of miRNAs, for example, was shown to respond to oxidative stress induced by hypoxic conditions in vitro, modelling conditions observed in AMD [11,41]. Let-7 miRNAs are highly expressed in retinal tissues and vascular endothelial cells [5,41]. Szemraj et al., 2015, proposed that Let-7 miRNAs promote pro-angiogenic processes in patients with wet AMD in response to these hypoxic conditions [11]. miR-27b also promotes angiogenesis via its regulation of Sprouty2 and semaphorin 6A target proteins. It is via these pathways that miR-27b has been implicated in the control of vascular endothelial sprouting in angiogenesis [21]. Ertekin et al. hypothesised that miR-27b acts as an early indicator of an angiogenic switch in AMD [21].

Grassmann et al., 2014, identified novel biomarkers for late-stage wet AMD through next-generation sequencing of circulating miRNAs in plasma from wet AMD patients [2]. Circulating miRNAs of 203 in number were identified, with miR-301a-3p, miR-361 expression notably shown to be significantly altered in patients compared with AMD-free controls [2]. Their role in cellular responses to ischaemic stress and damage was highlighted in this study, which implicated them in the control of TGF- and mTOR signalling pathways. [2]. Aberrant expression of miR-23a is also known to regulate gene expression in ischaemic conditions [42]. miR-23a was shown to regulate Fas expression in ARPE-19 cells, thereby promoting cell survival in the setting of oxidative stress [42]. Similarly, miR-874-3p has been shown to protect stroke patients from ischemic neuronal injury by inhibiting the pro-apoptotic factors BMF and BCL2L13 [43].

AMD has been shown to be associated with neurodegeneration, and many of the miRNAs identified in this study have been shown to have a role in neurodegenerative processes. Taking hsa-Let-7d-5p, this miRNA was suggested as a potential biomarker for Alzheimer’s disease [44]. This is particularly noteworthy given the link between AMD and Alzheimer’s [4,15] In addition to this, Shahriari et al. (2020) found that MITF expression in RPE cells was influenced by hsa-let-7a-5p, promoting RPE differentiation at the expense of neural differentiation [45]. miR-874-5p has also been implicated in influencing gene expression in in vitro neurodegenerative disease models. It has been associated with the promotion of neuronal damage in Parkinson’s disease (MPP+-triggered neuronal damage in SK-N-SH cells) via the miR-874-5p/ATG10 axis [46].

miRNAs are known to modulate several inflammatory processes, key in the pathogenesis of AMD, and a number of the miRNAs identified in this study have been explored as inflammatory mediators. miR23a-3p, for example, was shown to play a significant role in the modulation of pro-inflammatory TNF-α, IL-1β, IL-2, IL-4, IL-6, IL-12, and GM-CSF cytokines [14]. In addition to this, miR-19 family members, including miR-19a and miR19-b-1, were demonstrated to have a regulatory influence on inflammatory cells in lung cancer models. They accomplish this by modifying the expression of IFN-induced genes and MHC class I genes in human cancer cell lines [47]. Furthermore, miR-19b-1 suppresses the NF-B regulators A20/Tnfaip3, Rnf11, Fbxl11/Kdm2a, and Zbtb16, which promotes NF-B activity, a key promoter of inflammation [48].

Prior investigation into the role of circulating miRNAs in AMD by ElShelmani et al., 2021, explored the function of a miR-19a, miR-126, and miR-410 in vitro [49]. This current study found that at least one mature isoform of each of these miRNAs was expressed significantly higher in AMD patients than in healthy controls. The functional analysis of these miRNAs illustrated their roles in regulating VEGF signalling, apoptosis, and neurodegenerative pathways [49].

Interestingly, miR-410-5p and miR-626 did not show any notable difference in expression levels between AMD patients and healthy controls in this study, despite having been validated in AMD patients in previous studies. We hypothesise that these miRNAs might not be useful biomarkers in the context of Irish patients, perhaps because different populations might require different biomarker profiling. Further study would be required to explore and verify this. In contrast to its counterpart, miR-410-3p was significantly elevated in the AMD population. This highlights the diverging function of miRNAs after being processed into their mature forms and their differing functions in pathophysiological processes and warrants further investigation.

Notably, miRNAs shown in this study to be higher in dry AMD than wet AMD were previously shown in this author’s previous work to only have been increased in wet AMD [10]. This includes miR-let-7d, miR-27b and miR-874. Though not entirely clear, there may be a number of factors contributing to this discrepancy in results. First, the classification of AMD in this patient cohort (dry [non-neovascular] or wet [neovascular]) can be somewhat reductive, and there is ongoing work in clarifying whether or not AMD is really a spectrum of disease or a heterogenous group of disorders [50]. Second, the classification of patients as either dry or wet AMD in this study was conducted at a specific timepoint. Current clinical diagnostic methods fall short of identifying patients who are likely to develop neovascular changes. Further prospective research will be required to identify patients that might convert to wet AMD over time.

One potential limitation of this study is the small amount of miRNA collected from serum samples, meaning miRNAs were tested in single wells rather than in triplicate. This issue was overcome, however, by maximising our yield of good-quality miRNA from samples using the Qiagen extraction purification. Samples were quality controlled using the miRCURY LNA miRNA QC PCR panel, incorporating spike-in sequences to validate the quantity and quality of miRNA extracted.

Healthy volunteers were selected for this study. The study excluded patients and controls with co-existing ocular pathology and/or underlying systemic disorders such as diabetes, high blood pressure, and other inflammatory conditions which affect retinal health and could potentially skew results. Finding such patients in an elderly population proved difficult. Therefore, despite the fact that AMD patients were gender and age-matched with controls, there is potential for selection bias in this study. It is unclear whether this affected the variability of miRNA expression in either group.

In conclusion, we successfully validated the biomarker potential of a number of circulating miRNAs and characterised their expression in the context of an Irish population. We specifically focused on the mature miRNAs to better characterise miRNAs previously linked to AMD. The serum miRNAs that were identified show promise as potential serum biomarkers for the development of AMD. Interestingly, some of the miRNAs showed little to no change in expression from control to AMD patients, suggesting that these miRNAs may not be useful in the context of a white Irish population. Further investigation with a larger sample size in a more heterogenous population is needed. Additionally, candidate miRNAs identified should be assessed using prospective longitudinal studies to fully explore their usefulness as early indicators of disease and disease progression.

## 4. Materials and Methods

### 4.1. Case-Controlled Study Design

Clinically documented AMD patients and control blood donors were recruited at the Mater Misericordiae University Hospital (MMUH), Dublin. Ethics approval for the study was obtained from MMUH according to the tenets of the Declaration of Helsinki. All study participants were Caucasians from Ireland. All participants were over 60 years of age. Patients and controls with co-existing ocular pathology and/or underlying systemic diseases such as diabetic retinopathy, high blood pressure, and other inflammatory conditions were excluded from the study. Patients with AMD received a comprehensive eye examination by a clinician (DK) in the MMUH Eye Clinic and provided written informed consent. AMD patients from MMUH were defined and graded using the Age-Related Eye Disease Study (AREDS) macular degeneration classification system [51]. Blood specimens were collected, patient identifiers were removed, and the specimens were encoded to protect donor confidentiality. The study was designed to compare miRNA profiles in a number (*n* = 36) of control samples and dry/wet-AMD serum samples. AMD disease status was categorically based on fundus examination (dry or wet AMD). Study population characteristics are summarised in Table 3.

### 4.2. Human Serum Preparation

In order to collect serum samples, non-fasting blood specimens were collected with consent from each patient and control. From the collected blood samples, the red blood cells were allowed to clot naturally. These specimens were processed within 3–4 h of blood draw. The tubes were centrifuged at 400× *g* for 15 min. Following centrifugation, 1–1.5 mL of the serum was carefully removed, aliquoted and stored immediately at −80 °C until use.

### 4.3. RNA Extraction

Total RNA was extracted using a miRNeasy Serum/Plasma kit (Qiagen, Manchester, UK) according to the manufacturer’s protocol. First, 200 µL of patient serum was aliquoted into a 2 mL microcentrifuge tube, to which 60 µL of lysis buffer RPL (Qiagen, UK) was added, and 1 µL of UniSp2, UniSp4, and UniSp5 RNA spike-in mix was added to the lysis buffer. The tube was mixed vigorously by vortexing for 5 s and left at room temperature (15–25 °C) for 3 min to ensure complete lysis. Following this, 3.5 µL of miRNeasy Serum/Plasma Spike-In Control (Qiagen, UK) and lysis buffer was added and mixed thoroughly. 20 µL of buffer RPP was then added to precipitate the protein inhibitors in the serum. The tube was mixed vigorously by vortexing for 20 s to ensure precipitation and denaturation of serum proteins. This was left to stand at room temperature for a further 3 min. Tubes were then centrifuged at 12,000× *g* for 3 min using a centrifuge at room temperature to pellet the precipitated serum proteins.

The supernatant, which contains the RNA, was transferred to a fresh microcentrifuge tube and 1 volume of isopropanol was added to the tube. This allowed for suitable binding conditions for RNA molecules ≥ 18 nucleotides in length. The tube was mixed well by vortexing. The entire sample was then pipetted into a RNeasy UCP MinElute column (Qiagen, UK), within a 2 mL collection tube. The sample was then centrifuged for 15 s at ≥8000× *g*. The RNA bound to the matrix in the RNeasy UCP MinElute column. The flow-through in the collection tube was disposed of.

A number of washing steps were then undertaken, using 700 µL of Buffer RWT (Qiagen, UK), followed by 500 µL of Buffer RPE (Qiagen, UK) and finally 500 µL of 80% ethanol, each time centrifuging the sample and discarding the flow-through. For the wash steps using Buffer RWT and Buffer RPE, the tubes were centrifuged for 15 s at ≥8000× *g*, with the flow-through discarded. For the ethanol wash step the sample was centrifuged for 2 min at ≥8000× *g* (≥10,000 rpm), again disposing of the flow through.

Particular care was taken in removing the spin column from the collecting tube to ensure that the ethanol did not interfere with downstream reactions. Additionally, several steps were taken to dry the membrane of the RNeasy UCP MinElute spin column. First, the spin column was carefully removed from the collection tube, ensuring that the column did not touch the eluted flow-through containing ethanol. The column was placed into a new 2 mL collection tube and the membrane of the spin column was then dried by centrifugation at full speed for 5 min, this time with the lid open. Any and all flow-through was then discarded. Finally, to elute the total RNA, the RNeasy UCP MinElute spin column was transferred to a fresh 1.5 mL collection tube. Then, 20 µL of RNase-free water was pipetted directly to the centre of the column membrane and left to stand for 1 min. The sample was then centrifuged for 1 min at full speed, with isolated RNA contained in the flow-through.

### 4.4. Quality of Extracted RNA

The miRCURY LNA miRNA QC PCR Panel was used to analyse the robustness of the RNA isolation process and quality of isolated miRNA. The panel consists of 96-well PCR plates containing dried-down LNA PCR assays for one 10 µL real-time PCR reaction per well (Tables S2 and S3). UniSp2, UniSp4 and UniSp5 provide a control for the quality of the RNA isolation in any miRNA quantitative reverse-transcription PCR (qRT-PCR) experiment. miR-103-3p and miR-191-5p are well expressed in most tissues, whereas miR-451a and miR-23a-3p serve as a haemolysis marker and an internal control (Table S3).

Table S2 shows the assays included in the miRCURY LNA miRNA QC PCR Panel. Ahead of isolating the RNA from serum, 1 μL of RNA spike-in mix (UniSp2, UniSp4, UniSp5 RNA Spike-in mix, Qiagen, UK) was added to 60 μL of lysis buffer RPL. This mixture was added to samples after the addition of lysis buffer RPL but prior to the addition of precipitation buffer RPP, as described in the RNA isolation experiment above. Table S2 shows the application for the quality assay controls. UniSp2, UniSp4, and UniSp5 are present at a 100-fold concentration difference in the RNA Spike-in Kit, which results in approximately 5–7 Rq difference between the spike-ins. ΔRq (miR-23a and miR-451a) lower than 5 in human serum or plasma represents non-haemolyzed samples. If the ∆Rq is close to or higher than 7, there is an increased risk of haemolysis.

### 4.5. MicroRNA Quantitative Real-Time Polymerase Chain Reaction (qRT-PCR)

All qRT-PCR reactions were run in a LightCycler^®^ (RT-PCR) 96 instrument (Roche, UK), following the miRCURY LNA miRNA SYBR^®^ Green PCR kit (Qiagen, UK) protocol for exosomes, serum/plasma, and other biofluid samples. The custom serum focus microRNA panel was used for the validation set, which focuses on miRNAs of interest, and includes endogenous controls and RNA spike-in controls. Forty-two selected miRNAs and four endogenous control miRNAs were evaluated from serum in a 96-well plate with all the relevant controls (see Table S2 for the panel assay list).

RNA isolated from serum samples was reverse transcribed to cDNA using the miRCURY LNA RT kit, using a tabletop PCR machine for the 60-min, 42 °C incubation required for the reaction, as well as the 5-min-long 95 °C heat inactivation to terminate it. UniSp6 RNA spike-in controls were added to this reaction to ensure the quality of the experiment. Of the resultant cDNA samples, 5 μL was mixed with 195 μL of nuclease-free water, achieving a 1:40 dilution of cDNA suitable for the qRT-PCR reaction. The reaction mixture was then prepared by adding 5 μL of 2× miRCURY SYBR^®^ Green Master Mix, 1 μL RNase-free water, and 4 μL cDNA template. Then, the total 10 μL of reaction mixture was dispensed into each of the PCR wells. Following the 2-min initial heat activation of the DNA polymerase enzyme, the PCR reaction was run for a total of 40 cycles. LightCycler^®^ 96 Instrument Software Version 1.2 (Roche, UK) was used to obtain raw *C_T_* values. This information was exported (.txt format) for analysis.

### 4.6. Quality of qRT-PCR

The quality of the qRT-PCR step was measured again using the miRNeasy Serum/Plasma Spike-In Control (Qiagen, UK). One μL UniSp6 of RNA spike-in (Qiagen, UK) was added to samples during the reverse transcription reaction, as described above. RNA spike-in control UniSp6 (CP) was used to evaluate the RT reaction. The expression level of this assay indicated that the reverse transcription was successful; UniSp3 (IPC) evaluated the qPCR reaction which indicated good technical performance of the experiment.

### 4.7. Normalisation

For the present study, normalisation of serum miRNAs was performed based on the average *C_T_* of four normaliser assays, which included miR-323-3p, miR-324-3p, miR-423-3p, and miR-423-5p. miR-323-3p and miR-324-3p were recommended by Applied Biosystems (TLDA manufacturer) and used in previous serum/blood studies as endogenous controls [10]. miR-423-3p and miR-423-5p are stable endogenous candidates that were recommended by the miRNAs custom panel providers (Qiagen, UK [52,53]). The formula used to calculate the normalised values is:

Normalised *C_T_* (d *C_T_*) = assay *C_T_* (sample) − average *C_T_* (normaliser assays).

### 4.8. Statistical Analyses

Raw *C_T_* data (.txt format) were exported from LightCycler^®^ 96 Instrument Software (Roche, UK) and the following formula was used to calculate the fold change in C_T_. Calculations were performed using Excel (Microsoft):

ΔC_T_ = C_T_− C_T_ (endogenous control)

ΔΔC_T_ = ΔC_T_ − ΔC_T_ control

Fold change (RQ value) = 2 − ΔΔC_T_

Means and standard deviations were determined. The significance of difference in serum miRNA expression between cohorts (wet AMD, dry AMD, and control) was assessed using analysis of variance (ANOVA) with the Holm–Bonferroni method. In order to evaluate the presence of miRNAs specific to each type of AMD, post-hoc tests to interrogate the pairwise comparisons were used to determine whether there was a significant difference between the two samples: wet AMD vs. control and dry AMD vs. control.

## Figures and Tables

**Table 1:** miRNAs identified in the literature as being differentially expressed in AMD patients compared with healthy controls or implicated in AMD pathogenesis. All sample tissues are human, unless otherwise stated. PBNCs = Peripheral blood nucleated cells.

miRNA	Sample Type	AMD Type	Proposed Role in AMD Pathogenesis	Reference
**miR-374a**	Serum [10] Plasma [21]	Dry	Oxidative stress response [22] Neurodegeneration [22]	ElShelmani et al., 2020 [10] Ertekin et al., 2014 [21]
**miR-19a**	Serum [10]	Dry	Cell growth [10] Apoptosis [10] Angiogenesis [10]	ElShelmani et al., 2020 [10]
**miR-296-5p**	Serum [10]	Dry	Oxidative stress response [23]	ElShelmani et al., 2020 [10]
**miR-19b**	Serum [10]	Dry	Cell growth [10] Apoptosis [10] Angiogenesis [10]	ElShelmani et al., 2020 [10]
**miR-126**	Serum [4,10] PBNCs [14]	Dry + Wet	Inflammation [5,10] Angiogenesis [5,10] Apoptosis [10]	ElShelmani et al., 2020 [10] Litwińska et al., 2019 [14] Romano et al., 2017 [4]
**miR-16**	Plasma [24] PBNCs [14]	Dry + Wet	Apoptosis [24] Cell growth [24] Inflammation [14,24]	Litwińska et al., 2019 [14] Ulańczyk et al., 2019 [24]
**miR-486-5p**	Serum [10] Serum exosomes [8]	Dry + Wet	Angiogenesis [8] Inflammation [8] Cell growth [8] Apoptosis [8]	ElShelmani et al., 2020 [10] Elbay et al., 2019 [8]
**hsa-miR-155**	Serum [4] Plasma [21,24] PBNCs [14] Retina [25]	Dry + Wet	Inflammation [4,5,6] Angiogenesis [5,6] Apoptosis [4]	Litwińska et al., 2019 [14] Ulańczyk et al., 2019 [24] Romano et al., 2017 [4] Ertekin et al., 2017 [21] Pogue et al., 2018 [25]
**hsa-miR-626**	Serum exosomes [8]	Dry + Wet	Neurodegeneration [8]	Elbay et al., 2019 [8]
**hsa-miR-9**	Serum [4]	Dry + Wet	Inflammation [5] Oxidative stress response [5]	Romano et al., 2017 [4]
**hsa-miR-23a**	Serum [4] Plasma [24] PBNCs [14] Mouse retinal tissue [26]	Dry + Wet	Oxidative stress response [5] Angiogenesis [5]	Litwińska et al., 2019 [14] Ulańczyk et al., 2019 [24] Romano et al., 2017 [4] Zhou et al., 2011 [24]
**hsa-miR-34a**	Serum [4] In vitro retinal pigment epithelium [27] Mouse retinal tissue [28]	Dry + Wet	Oxidative stress response [5] Cell growth [27] Cell proliferation [27]	Romano et al., 2017 [4] Smit-McBride et al., 2014 [28] Hou et al., 2013 [27]
**miR-874**	Serum [10]	Wet	Neurodegeneration [29]	ElShelmani et al., 2020 [10]
**miR-132**	Serum [10] Serum exosomes [8]	Wet	Angiogenesis [5,8] Cell proliferation [8] Angiogenesis [8]	ElShelmani et al., 2020 [10] Elbay et al., 2019 [8]
**miR-27b**	Serum [10] Plasma [21]	Wet	Angiogenesis [5,21]	ElShelmani et al., 2020 [10] Ertekin et al., 2014 [21]
**miR-25**	Serum [10] Plasma [21] Whole blood [9] Rat retinal tissue [30]	Wet	Oxidative stress response [30]	ElShelmani et al., 2020 [10] Ren et al., 2017 [9] Zhang et al., 2017 [30] Ertekin et al., 2014 [21]
**miR-146a**	Serum [4,10] Plasma [18] PBNCs [14] Vitreous humour [31] Retina [25]	Wet	Inflammation [4,5,6] Oxidative stress response [6] Neurodegeneration [6] Angiogenesis [6]	ElShelmani et al., 2020 [10] Litwińska et al., 2019 [14] Ulańczyk et al., 2019 [24] Romano et al., 2017 [4] Ménard et al., 2016 [31] Pogue et al., 2018 [25]
**miR-410**	Serum [10] Plasma [21]	Wet	Inflammation [10]	ElShelmani et al., 2020 [10] Ertekin et al., 2014 [21]
**hsa-miR-125b**	Retinoblastoma cell lines [32] Retina [25]	Wet	Inflammation [5] Angiogenesis [6]	Pogue et al., 2018 [25] Bai et al., 2016 [32]
**hsa-miR-27a**	Whole blood [9] Serum [4] Mouse retinal tissue [26]	Wet	Angiogenesis [5] Neurodegeneration [4] Inflammation [4]	Ren et al., 2017 [9] Romano et al., 2017 [4] Zhou et al., 2011 [26]
**hsa-miR-93**	Mouse retinal tissue [1] Plasma [24]	Wet	Angiogenesis [1,5,24]	Wang et al., 2016 [4] Ulańczyk et al., 2019 [24]
**hsa-miR-301-3p**	Serum [2]	Wet	Angiogenesis [2]	Grassmann et al., 2014 [2]
**hsa-miR-361-5p**	Serum [2]	Wet	Angiogenesis [2]	Grassmann et al., 2014 [2]
**hsa-miR-424-5p**	Serum [2,11]	Wet	Angiogenesis [2]	Grassmann et al., 2014 [2] Szemraj et al., 2015 [11]
**hsa-let-7a-5p**	Serum [11]	Wet	Angiogenesis [11]	Szemraj et al., 2015 [11]
**hsa-let-7d-5p**	Serum [11]	Wet	Angiogenesis [11]	Szemraj et al., 2015 [11]
**hsa-let-7b-5p**	Serum [11]	Wet	Angiogenesis [11]	Szemraj et al., 2015 [11]
**miR-200b**	Mouse retinal tissue [33] Rat retinal tissue [34]	Wet	Oxidative stress response [33,34] Angiogenesis [34]	Murray et al., 2013 [33] McArthur et al., 2011 [34]
**miR-205-5p**	Serum [7] In vitro retinal pigment epithelium [35]	Wet	Oxidative stress response [35] Angiogenesis [7,35]	Oltra et al., 2020 [35] Blasiak et al., 2019 [7]

**Table 2:** miRNA names, *p*-values from ANOVA of dry, wet and control samples, and post-hoc tests comparing dry vs. control and wet vs. control samples. *p*-values: * ≤ 0.05, ** ≤ 0.01, *** ≤ 0.001.

miRNA	ANOVA *p*-Value	*p*-Value Adjusted for Multiple Tests	Post-Hoc Test	
			Dry vs. Control	Wet vs. Control
**let-7a-5p**	0.0000023 ***	0.0000915 ***	4.57E-08 ***	4.04E-04 ***
**let-7b-5p**	0.0000338 ***	0.0011159 **	0.000000102 ***	5.46E-05 ***
**let-7d-5p**	0.0000002 ***	0.0000084 ***	5.42E-09 ***	3.30E-04 ***
**miR-125b-5p**	0.0041234 **	0.0536036	0.001766642 **	0.000150437 ***
**miR-155-5p**	0.0002756 ***	0.0064747 **	0.000000316 ***	7.85E-04 ***
**miR-23a-3p**	0.0000055 ***	0.0002102 ***	8.03E-08 ***	1.12E-02 *
**miR-27a-3p**	0.0000339 ***	0.0011159 **	3.67E-09 ***	1.53E-04 ***
**miR-301a-3p**	0.0000173 ***	0.0006216 ***	0.000000121 ***	1.74E-03 **
**miR-34a-3p**	0.0029547 **	0.0472756 *	0.001283794 **	0.001283794 **
**miR-361-5p**	0.0000003 ***	0.000013 ***	2.93E-09 ***	1.27E-04 ***
**miR-424-5p**	0.0134416 *	0.0997292	0.000240525 ***	0.002441783 **
**miR-626**	0.0282197 *	0.1128788	1	0.0241612 *
**miR-9-5p**	0.012466 1 *	0.0997292	0.005665279 **	0.078181678
**miR-93-5p**	0.0000965 ***	0.0027007 **	0.00000149 ***	7.59E-04 ***
**miR-126-3p**	0.0000879 ***	0.0026358 **	8.65E-08 ***	1.22E-03**
**miR-132-3p**	0.0000976 ***	0.0027007 **	0.00000751 ***	1.62E-04 ***
**miR-146a-5p**	0.0000308 ***	0.0010487 **	0.000000118 ***	5.47E-03 **
**miR-16-1-3p**	0.006405 **	0.0659499	0.000417055 ***	0.004765244 **
**miR-19a-3p**	0.0009344 ***	0.0177531 *	0.000000344 ***	1.14E-04 ***
**miR-19b-3p**	0.0000817 ***	0.0025317 **	7.75E-08 ***	2.72E-04 ***
**miR-25-3p**	0.0003701 ***	0.0077725 **	0.0000263 ***	1.04E-02 *
**miR-27b-3p**	0.0000016 ***	0.0000658 ***	2.46E-08 ***	1.00E-02 *
**miR-296-5p**	0.0000902 ***	0.0026358 **	0.00000361 ***	1.42E-04 ***
**miR-374a-3p**	0.0002002 ***	0.0052054 **	0.0000189 ***	2.24E-02 *
**miR-410-3p**	0.0002989 ***	0.0065768 **	0.00000215 ***	3.08E-02 *
**miR-486-5p**	0.0026621**	0.0452558 *	0.000196392 ***	0.012940657 *
**miR-874-3p**	0.0000208 ***	0.0007281 ***	0.000000903 ***	7.41E-05 ***
**miR-200b-3p**	0.0059954 **	0.0659499	0.000513111 ***	0.048144232 *
**hsa-miR-205-5p**	0.4166033	0.8332066	0.01844464 *	0.04330408 *
**miR-23a-5p**	0.017179 *	0.1030738	0.002440702 **	0.002440702 **
**miR-27a-5p**	0.0094173 **	0.0847555	0.001859376 **	0.019768118 *
**miR-34a-5p**	0.0002132 ***	0.0053307 **	0.0000367 ***	3.67E-05 ***
**miR-126-5p**	0.0002698 ***	0.0064747 **	0.00000375 ***	6.31E-04 ***
**miR-132-5p**	0.0839077	0.251723	0.09591552	0.43207043
**miR-16-5p**	0.0029939 **	0.0472756 *	0.00000267 ***	1.69E-04 ***
**miR-19a-5p**	0.0047982 **	0.0575782	0.0000288 ***	2.44E-03 **
**miR-25-5p**	0.0215507 *	0.1077534	0.004209305 **	0.042136988 *
**miR-27b-5p**	0.0030667 **	0.0472756 *	0.002349743 **	0.113657163
**miR-374a-5p**	0.0007715 ***	0.01543 *	0.000000704 ***	4.05E-03 **
**miR-410-5p**	0.4354663	0.8332066	0.9400418	0.5086931
**miR-874-5p**	0.0011751 **	0.0211526 *	0.000117408 ***	0.028667993 *
**miR-19b-1-5p**	0.0000068 ***	0.0002523 ***	0.00000196 ***	9.66E-02

**Table 3:** Patient characteristics (*n* = 36).

Characteristic		Control (*n* = 10)	Dry (*n* = 12)	Wet (*n* = 14)
Age	Mean	72	70	75
	60–69	*n* = 2	*n* = 9	*n* = 5
	70–79	*n* = 8	*n* = 3	*n* = 8
	>80	*n* = 0	*n* = 0	*n* = 1
Gender	Female	*n* = 6	*n* = 7	*n* = 9
	Male	*n* = 4	*n* = 5	*n* = 5

## Data Availability

The data is available within the text and the supplementary section. Also, available at Next Generation Sequencing laboratory, Mater Misericordiae University Hospital, Dublin, Ireland.

## Supplementary Materials

The following are available online at https://www.mdpi.com/article/10.3390/ijms222212321/s1.
