# Supplementary material for: Differential Circulating MicroRNA Expression in Age-Related Macular Degeneration
Source: Int J Mol Sci. 2021 Nov 15;22(22):12321. doi: 10.3390/ijms222212321 (PMC8625913; doi:10.3390/ijms222212321)
Supplement: Supplementary file 1 [file ijms-22-12321-s001.zip › ijms-1424057-supplementary.pdf]

Supplementary Materials

Table S1: Assays included in the miRCURY LNA miRNA QC PCR Panel.

	Assay (common name*)	Human, miRbase v20	Mouse, miRbase v20
miRNA assay	miR-103	hsa-miR-103a-3p	mmu-miR-103-3p
miRNA assay	miR-191	mmu-miR-191-5p	mmu-miR-191-5p
miRNA assay	miR-451	hsa-miR-451a	mmu-451
miRNA assay	miR-23a	hsa-miR-23a-3p	mmu-miR-23a-3p
RT assay	UniSp6, v2*	–	–
Spike-in assay	UniSp2	–	–
Spike-in assay	UniSp4	–	–
Spike-in assay	UniSp5, v2*	–	–
RT assay	cel-miR-39-3p	–	–
Inter-plate calibrator / PCR control	UniSp3	–	–
miRNA assay	miR-124	hsa-miR-124-3p	mmu-miR-124-3p
miRNA assay	miR-30c	hsa-miR-30c-5p	mmu-miR-30c-5p

* In the species rno, cfa and mml (rat, dog and rhesus), the common names are used for these miRNAs.
† New, optimized UniSp5 and UniSp6 assays designed for V4 panels target same RNA template as previous assays.

Table S2: Applications for the quality assay controls.

Assay name	Applications for biofluid samples
UniSp2, UniSp4 and UniSp5	• Three different concentrations<br>• Add to lysis buffer during RNA purification<br>• Use to monitor RNA isolation efficiency
UniSp6 and cel-miR-39-3p	• Different concentrations<br>• Add to cDNA synthesis reaction<br>• Check for RT and PCR inhibitors
UniSp3	• Template and primers present in PCR panels<br>• Independent PCR monitoring<br>• Use as inter-plate calibrator (IPC)
miR-451a and miR-23a-3p	• Unique hemolysis indicator<br>• Check ΔC_q (miR-23a-3p – miR-451a)
miR-30c-5p, miR-103a-3p, miR-124-3p and miR-191-5p	• Biologically relevant, endogenous miRNAs<br>• Should be present in RNA from serum/plasma, urine or CSF and some other biofluids<br>• Use for general sample quality check

Table S3 A: Panel assay list.

	A	B	C	D	E	F	G	H
**1**	hsa-let-7a-5p	hsa-miR-27a-3p	hsa-miR-9-5p	hsa-miR-19a-3p	hsa-miR-410-3p	hsa-miR-27a-5p	hsa-miR-25-5p	hsa-miR-324-3p
**2**	hsa-let-7b-5p	hsa-miR-301a-3p	hsa-miR-93-5p	hsa-miR-19b-3p	hsa-miR-486-5p	hsa-miR-34a-5p	hsa-miR-27b-5p	hsa-miR-423-3p
**3**	hsa-let-7d-5p	hsa-miR-34a-3p	hsa-miR-126-3p	hsa-miR-25-3p	hsa-miR-874-3p	hsa-miR-126-5p	hsa-miR-374a-5p	hsa-miR-423-5p
**4**	hsa-miR-125b-5p	hsa-miR-361-5p	hsa-miR-132-3p	hsa-miR-27b-3p	hsa-miR-200b-3p	hsa-miR-132-5p	hsa-miR-410-5p	hsa-miR-19b-1-5p
**5**	hsa-miR-155-5p	hsa-miR-424-5p	hsa-miR-146a-5p	hsa-miR-296-5p	hsa-miR-205-5p	hsa-miR-16-5p	hsa-miR-874-5p	UniSp3
**6**	hsa-miR-23a-3p	hsa-miR-626	hsa-miR-16-1-3p	hsa-miR-374a-3p	hsa-miR-23a-5p	hsa-miR-19a-5p	hsa-miR-323a-3p	UniSp6
**7**	hsa-let-7a-5p	hsa-miR-27a-3p	hsa-miR-9-5p	hsa-miR-19a-3p	hsa-miR-410-3p	hsa-miR-27a-5p	hsa-miR-25-5p	hsa-miR-324-3p
**8**	hsa-let-7b-5p	hsa-miR-301a-3p	hsa-miR-93-5p	hsa-miR-19b-3p	hsa-miR-486-5p	hsa-miR-34a-5p	hsa-miR-27b-5p	hsa-miR-423-3p
**9**	hsa-let-7d-5p	hsa-miR-34a-3p	hsa-miR-126-3p	hsa-miR-25-3p	hsa-miR-874-3p	hsa-miR-126-5p	hsa-miR-374a-5p	hsa-miR-423-5p
**10**	hsa-miR-125b-5p	hsa-miR-361-5p	hsa-miR-132-3p	hsa-miR-27b-3p	hsa-miR-200b-3p	hsa-miR-132-5p	hsa-miR-410-5p	hsa-miR-19b-1-5p
**11**	hsa-miR-155-5p	hsa-miR-424-5p	hsa-miR-146a-5p	hsa-miR-296-5p	hsa-miR-205-5p	hsa-miR-16-5p	hsa-miR-874-5p	UniSp3
**12**	hsa-miR-23a-3p	hsa-miR-626	hsa-miR-16-1-3p	hsa-miR-374a-3p	hsa-miR-23a-5p	hsa-miR-19a-5p	hsa-miR-323a-3p	UniSp6

	1	2	3	4	5	6	7	8	9	10	11	12	
**A**	hsa-let-7a-5p	hsa-let-7b-5p	hsa-let-7d-5p	hsa-miR-125b-5p	hsa-miR-155-5p	hsa-miR-23a-3p	hsa-let-7a-5p	hsa-let-7b-5p	hsa-let-7d-5p	hsa-miR-125b-5p	hsa-miR-155-5p	hsa-miR-23a-3p	
**B**	hsa-miR-27a-3p	hsa-miR-301a-3p	hsa-miR-34a-3p	hsa-miR-361-5p	hsa-miR-424-5p	hsa-miR-626	hsa-miR-27a-3p	hsa-miR-301a-3p	hsa-miR-34a-3p	hsa-miR-361-5p	hsa-miR-424-5p	hsa-miR-626	
**C**	hsa-miR-9-5p	hsa-miR-93-5p	hsa-miR-126-3p	hsa-miR-132-3p	hsa-miR-146a-5p	hsa-miR-16-1-3p	hsa-miR-9-5p	hsa-miR-93-5p	hsa-miR-126-3p	hsa-miR-132-3p	hsa-miR-146a-5p	hsa-miR-16-1-3p	
**D**	hsa-miR-19a-3p	hsa-miR-19b-3p	hsa-miR-25-3p	hsa-miR-27b-3p	hsa-miR-296-5p	hsa-miR-374a-3p	hsa-miR-19a-3p	hsa-miR-19b-3p	hsa-miR-25-3p	hsa-miR-27b-3p	hsa-miR-296-5p	hsa-miR-374a-3p	
**E**	hsa-miR-410-3p	hsa-miR-486-5p	hsa-miR-874-3p	hsa-miR-200b-3p	hsa-miR-205-5p	hsa-miR-23a-5p	hsa-miR-410-3p	hsa-miR-486-5p	hsa-miR-874-3p	hsa-miR-200b-3p	hsa-miR-205-5p	hsa-miR-23a-5p	
**F**	hsa-miR-27a-5p	hsa-miR-34a-5p	hsa-miR-126-5p	hsa-miR-132-5p	hsa-miR-16-5p	hsa-miR-19a-5p	hsa-miR-27a-5p	hsa-miR-34a-5p	hsa-miR-126-5p	hsa-miR-132-5p	hsa-miR-16-5p	hsa-miR-19a-5p	
**G**	hsa-miR-25-5p	hsa-miR-27b-5p	hsa-miR-374a-5p	hsa-miR-410-5p	hsa-miR-874-5p	hsa-miR-323a-3p	hsa-miR-25-5p	hsa-miR-27b-5p	hsa-miR-374a-5p	hsa-miR-410-5p	hsa-miR-874-5p	hsa-miR-323a-3p	
**H**	hsa-miR-324-3p	hsa-miR-423-3p	hsa-miR-423-5p	hsa-miR-19b-1-5p	UniSp 3	UniSp 6	hsa-miR-324-3p	hsa-miR-423-3p	hsa-miR-423-5p	hsa-miR-19b-1-5p	UniSp 3	UniSp 6	

Table S3 B: miRNAs panel sequences.

**Row**	**Column**	**miRNA ID**	**Target sequence**	**Order in 96 (for sorting by row)**	**Order in 96 (for sorting by column)**

A	1	hsa-let-7a-5p	UGAGGUAGUAGGUUGUAUAGUU	1	1
A	2	hsa-let-7b-5p	UGAGGUAGUAGGUUGUGUGGUU	2	9
A	3	hsa-let-7d-5p	AGAGGUAGUAGGUUGCAUAGUU	3	17
A	4	hsa-miR-125b-5p	UCCCUGAGACCCUAACUUGUGA	4	25
A	5	hsa-miR-155-5p	UUAAUGCUAAUCGUGAUAGGGGU	5	33
A	6	hsa-miR-23a-3p	AUCACAUUGCCAGGGAUUUCC	6	41
A	7	hsa-let-7a-5p	UGAGGUAGUAGGUUGUAUAGUU	7	49
A	8	hsa-let-7b-5p	UGAGGUAGUAGGUUGUGUGGUU	8	57
A	9	hsa-let-7d-5p	AGAGGUAGUAGGUUGCAUAGUU	9	65
A	10	hsa-miR-125b-5p	UCCCUGAGACCCUAACUUGUGA	10	73
A	11	hsa-miR-155-5p	UUAAUGCUAAUCGUGAUAGGGGU	11	81
A	12	hsa-miR-23a-3p	AUCACAUUGCCAGGGAUUUCC	12	89
B	1	hsa-miR-27a-3p	UUCACAGUGGCUAAGUUCCGC	13	2
B	2	hsa-miR-301a-3p	CAGUGCAAUAGUAUUGUCAAAGC	14	10
B	3	hsa-miR-34a-3p	CAAUCAGCAAGUAUACUGCCCU	15	18
B	4	hsa-miR-361-5p	UUAUCAGAAUCUCCAGGGGUAC	16	26
B	5	hsa-miR-424-5p	CAGCAGCAAUUCAUGUUUUGAA	17	34
B	6	hsa-miR-626	AGCUGUCUGAAAAUGUCUU	18	42
B	7	hsa-miR-27a-3p	UUCACAGUGGCUAAGUUCCGC	19	50
B	8	hsa-miR-301a-3p	CAGUGCAAUAGUAUUGUCAAAGC	20	58
B	9	hsa-miR-34a-3p	CAAUCAGCAAGUAUACUGCCCU	21	66
B	10	hsa-miR-361-5p	UUAUCAGAAUCUCCAGGGGUAC	22	74

B	11	hsa-miR-424-5p	CAGCAGCAAUUCAUGUUUUGAA	23	82
B	12	hsa-miR-626	AGCUGUCUGAAAAUGUCUU	24	90
C	1	hsa-miR-9-5p	UCUUUGGUUAUCUAGCUGUAUGA	25	3
C	2	hsa-miR-93-5p	CAAAGUGCUGUUCGUGCAGGUAG	26	11
C	3	hsa-miR-126-3p	UCGUACCGUGAGUAAUAAUGCG	27	19
C	4	hsa-miR-132-3p	UAACAGUCUACAGCCAUGGUCG	28	27
C	5	hsa-miR-146a-5p	UGAGAACUGAAUUCCAUGGGUU	29	35
C	6	hsa-miR-16-1-3p	CCAGUAUUAACUGUGCUGCUGA	30	43
C	7	hsa-miR-9-5p	UCUUUGGUUAUCUAGCUGUAUGA	31	51
C	8	hsa-miR-93-5p	CAAAGUGCUGUUCGUGCAGGUAG	32	59
C	9	hsa-miR-126-3p	UCGUACCGUGAGUAAUAAUGCG	33	67
C	10	hsa-miR-132-3p	UAACAGUCUACAGCCAUGGUCG	34	75
C	11	hsa-miR-146a-5p	UGAGAACUGAAUUCCAUGGGUU	35	83
C	12	hsa-miR-16-1-3p	CCAGUAUUAACUGUGCUGCUGA	36	91
D	1	hsa-miR-19a-3p	UGUGCAAAUCUAUGCAAAACUGA	37	4
D	2	hsa-miR-19b-3p	UGUGCAAAUCCAUGCAAAACUGA	38	12
D	3	hsa-miR-25-3p	CAUUGCACUUGUCUCGGUCUGA	39	20
D	4	hsa-miR-27b-3p	UUCACAGUGGCUAAGUUCUGC	40	28
D	5	hsa-miR-296-5p	AGGGCCCCCCCUCAAUCCUGU	41	36
D	6	hsa-miR-374a-3p	CUUAUCAGAUUGUAUUGUAAUU	42	44
D	7	hsa-miR-19a-3p	UGUGCAAAUCUAUGCAAAACUGA	43	52
D	8	hsa-miR-19b-3p	UGUGCAAAUCCAUGCAAAACUGA	44	60

D	9	hsa-miR-25-3p	CAUUGCACUUGUCUCGGUCUGA	45	68
D	10	hsa-miR-27b-3p	UUCACAGUGGCUAAGUUCUGC	46	76
D	11	hsa-miR-296-5p	AGGGCCCCCCCUCAAUCCUGU	47	84
D	12	hsa-miR-374a-3p	CUUAUCAGAUUGUAUUGUAAUU	48	92
E	1	hsa-miR-410-3p	AAUAUAACACAGAUGGCCUGU	49	5
E	2	hsa-miR-486-5p		50	13
E	3	hsa-miR-874-3p	CUGCCCUGGCCCGAGGGACCGA	51	21
E	4	hsa-miR-200b-3p	UAAUACUGCCUGGUAAUGAUGA	52	29
E	5	hsa-miR-205-5p	UCCUUCAUUCCACCGGAGUCUG	53	37
E	6	hsa-miR-23a-5p	GGGGUUCCUGGGGAUGGGAUUU	54	45
E	7	hsa-miR-410-3p	AAUAUAACACAGAUGGCCUGU	55	53
E	8	hsa-miR-486-5p		56	61
E	9	hsa-miR-874-3p	CUGCCCUGGCCCGAGGGACCGA	57	69
E	10	hsa-miR-200b-3p	UAAUACUGCCUGGUAAUGAUGA	58	77
E	11	hsa-miR-205-5p	UCCUUCAUUCCACCGGAGUCUG	59	85
E	12	hsa-miR-23a-5p	GGGGUUCCUGGGGAUGGGAUUU	60	93
F	1	hsa-miR-27a-5p	AGGGCUUAGCUGCUUGUGAGCA	61	6
F	2	hsa-miR-34a-5p	UGGCAGUGUCUUAGCUGGUUGU	62	14
F	3	hsa-miR-126-5p	CAUUAUUACUUUUGGUACGCG	63	22
F	4	hsa-miR-132-5p	ACCGUGGCUUUCGAUUGUUACU	64	30
F	5	hsa-miR-16-5p	UAGCAGCACGUAAAUAUUGGCG	65	38
F	6	hsa-miR-19a-5p	AGUUUUGCAUAGUUGCACUACA	66	46

F	7	hsa-miR-27a-5p	AGGGCUUAGCUGCUUGUGAGCA	67	54
F	8	hsa-miR-34a-5p	UGGCAGUGUCUUAGCUGGUUGU	68	62
F	9	hsa-miR-126-5p	CAUUAUUACUUUUGGUACGCG	69	70
F	10	hsa-miR-132-5p	ACCGUGGCUUUCGAUUGUUACU	70	78
F	11	hsa-miR-16-5p	UAGCAGCACGUAAAUAUUGGCG	71	86
F	12	hsa-miR-19a-5p	AGUUUUGCAUAGUUGCACUACA	72	94
G	1	hsa-miR-25-5p	AGGCGGAGACUUGGGCAAUUG	73	7
G	2	hsa-miR-27b-5p	AGAGCUUAGCUGAUUGGUGAAC	74	15
G	3	hsa-miR-374a-5p	UUAUAAUACAACCUGAUAAGUG	75	23
G	4	hsa-miR-410-5p	AGGUUGUCUGUGAUGAGUUCG	76	31
G	5	hsa-miR-874-5p	CGGCCCCACGCACCAGGGUAAGA	77	39
G	6	hsa-miR-323a-3p	CACAUUACACGGUCGACCUCU	78	47
G	7	hsa-miR-25-5p	AGGCGGAGACUUGGGCAAUUG	79	55
G	8	hsa-miR-27b-5p	AGAGCUUAGCUGAUUGGUGAAC	80	63
G	9	hsa-miR-374a-5p	UUAUAAUACAACCUGAUAAGUG	81	71
G	10	hsa-miR-410-5p	AGGUUGUCUGUGAUGAGUUCG	82	79
G	11	hsa-miR-874-5p	CGGCCCCACGCACCAGGGUAAGA	83	87
G	12	hsa-miR-323a-3p	CACAUUACACGGUCGACCUCU	84	95
H	1	hsa-miR-324-3p	ACUGCCCCAGGUGCUGCUGG	85	8
H	2	hsa-miR-423-3p	AGCUCGGUCUGAGGCCCCUCAGU	86	16
H	3	hsa-miR-423-5p	UGAGGGGCAGAGAGCGAGACUUU	87	24
H	4	hsa-miR-19b-	AGUUUUGCAGGUUUGCAUCC	88	32

		1-5p	AGC		
H	5	UniSp3		89	40
H	6	UniSp6		90	48
H	7	hsa-miR-324-3p	ACUGCCCCAGGUGCUGCUGG	91	56
H	8	hsa-miR-423-3p	AGCUCGGUCUGAGGCCCCUCAGU	92	64
H	9	hsa-miR-423-5p	UGAGGGGCAGAGAGCGAGACUUU	93	72
H	10	hsa-miR-19b-1-5p	AGUUUUGCAGGUUUGCAUCCAGC	94	80
H	11	UniSp3		95	88
H	12	UniSp6		96	96

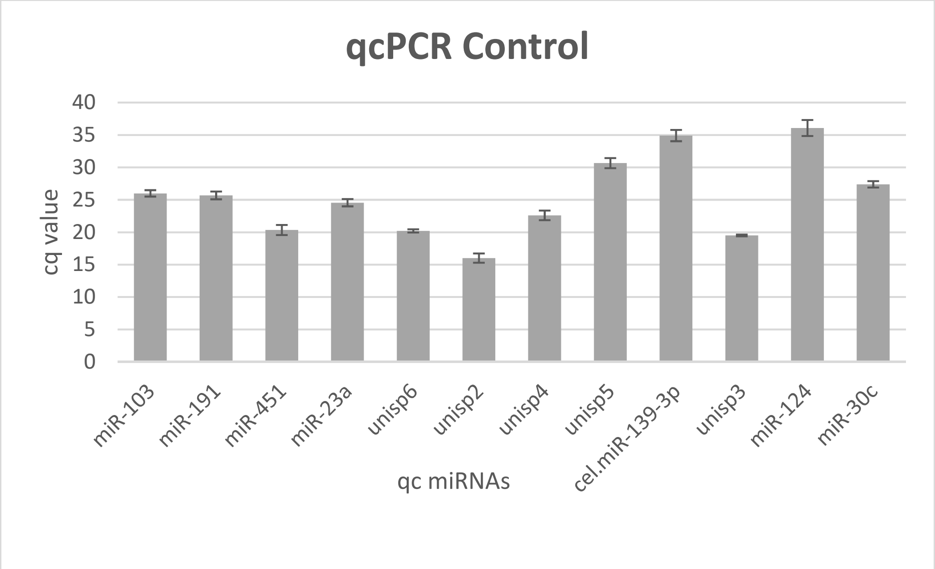

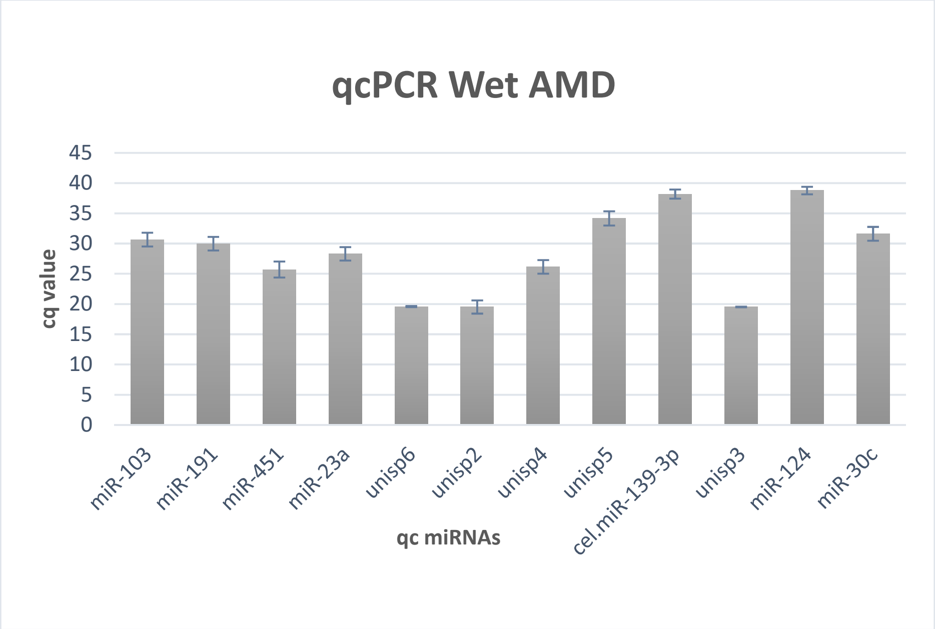

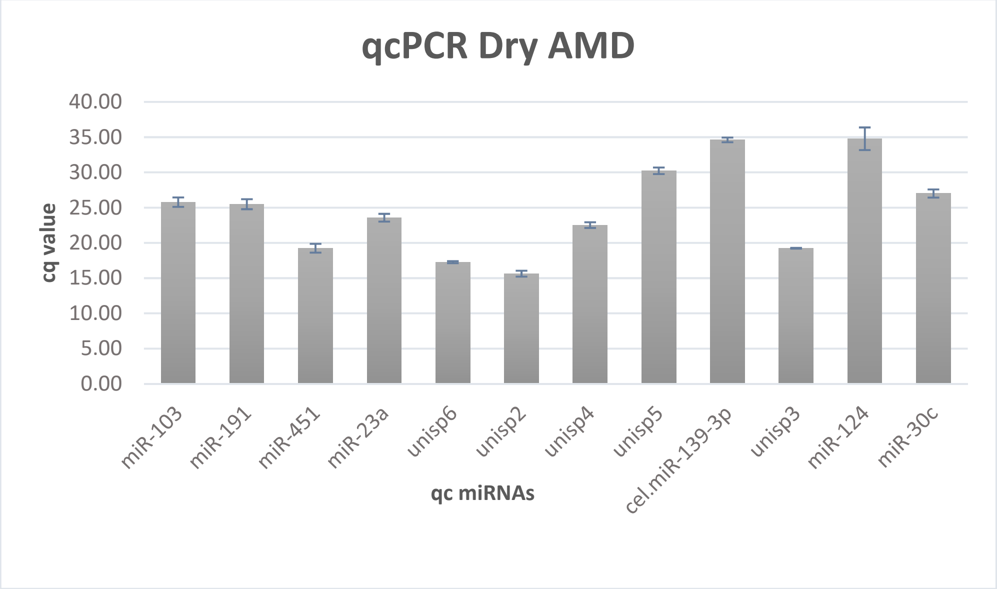

Figure S1: The miRCURY LNA miRNA QC PCR Pane results, miR-451a, miR-23a-3p, UniSp2, UniSp4, and UniSp5 RNA spike-in.

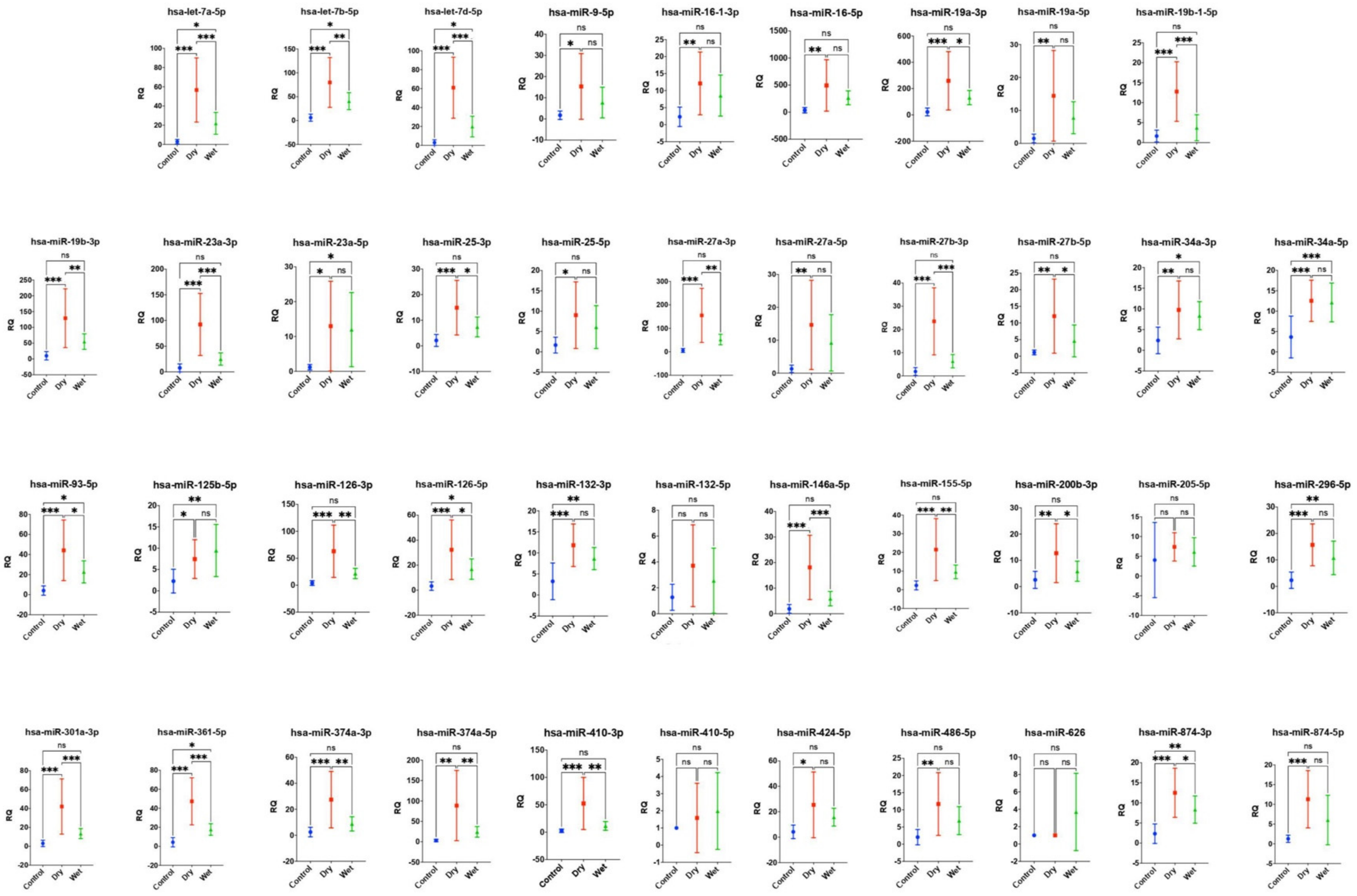

Figure S2. miRNA expression levels (Relative Quantification (RQ)) for three patient groups including the data points outliers. Control = patients free from AMD and diseases with similar symptoms; Dry (atrophic) = patients exhibiting the atrophic form of AMD; Wet (neovascular) = patients exhibiting the neovascular form of AMD. P-values: * ≤ 0.05, ** ≤ 0.01, *** ≤ 0.001.
